# Digital Health Literacy and Tool Adoption in Postoperative Care in a Safety-Net Hospital Population: Mixed Methods Study

**DOI:** 10.2196/75496

**Published:** 2026-02-23

**Authors:** Christopher Awad, Alexander Jakub Martinek, Chunhao Zou, Rosalind Byrd, Hui Jean Ooi, Kimberly Do, Henry Young, Bhavin Adhyaru, Muhammed Idris, Rosa I Arriaga

**Affiliations:** 1 Department of Emergency Medicine, The Ohio State University Wexner Medical Center, Columbus, OH, United States; 2 Clinical Research Center, Morehouse School of Medicine, 720 Westview Drive SW, Atlanta, GA, 30310, United States, 14702238755; 3 Department of Computer Science, Georgia Institute of Technology, Atlanta, GA, United States; 4 Division of General Internal Medicine, School of Medicine, Emory University, Atlanta, GA, United States; 5 College of Computing, Northeastern University, Boston, MA, United States

**Keywords:** patient navigation, digital health, mobile health app, postsurgical care, user experience, eHealth literacy, patient self-management, health informatics, digital intervention, human-computer interaction, usability testing, health care technology acceptance​​​​​​​​​​​​​​​​

## Abstract

**Background:**

Digital health tools are increasingly prevalent in postoperative care management, yet limited research exists on digital health literacy and tool adoption among safety-net hospital populations. Understanding these factors is crucial for developing effective digital health solutions for historically underserved communities.

**Objective:**

This study aimed to evaluate digital health literacy, assess technology adoption readiness, and examine the relationship between patient-reported capabilities and demographic factors in a postoperative care context at a safety-net hospital.

**Methods:**

We conducted a mixed methods study with 71 postoperative patients and 29 health care providers at a safety-net hospital. Participants completed a modified eHealth Literacy Scale (eHEALS) assessment and a demographic questionnaire, followed by usability testing of PocketDoc, a digital health prototype. The modified 7-item eHEALS demonstrated adequate internal consistency (Cronbach α=0.77). Qualitative data from think-aloud protocols during usability testing were collected for future analysis. This study focused on quantitative assessments of digital health literacy (using the modified eHEALS on a 5-point Likert scale) and technology adoption readiness (via usability metrics on a 10-point Likert scale) analyzed using nonparametric statistical tests. Correlations between demographic factors and digital health literacy were examined using Spearman rank-order correlation.

**Results:**

Despite common assumptions about technology barriers in safety-net populations, 69% (49/71) of patients reported high confidence (score of ≥3 on a 5-point scale) in finding health resources online, and 61% (43/71) expressed confidence in using the internet for health-related questions. However, only 49% (35/71) felt confident in using digital resources for health decision-making. Digital health literacy scores did not correlate with age or educational level, although 79% (56/71) of patients reported ≥10 years of digital device experience. Both patients and health care providers rated PocketDoc highly for ease of use (median 10, IQR 8-10) and task intuitiveness (median 10, IQR 8-10). Patients’ confidence in finding and using health resources online positively correlated with interface satisfaction (ρ=0.262-0.304 and ρ=0.010-0.027, respectively).

**Conclusions:**

Our exploratory findings from 100 participants suggest that digital health tools may be more feasible in safety-net settings than previously considered, although the sample size and single-site design limit generalizability. However, the gap between patients’ ability to find health resources (49/71, 69% confident) and their confidence in using these resources for health decision-making (35/71, 49% confident) highlights the need for targeted support in translating digital capabilities to health management skills.

## Introduction

Postoperative care increasingly relies on digital health tools to support patient recovery, manage symptoms, and facilitate physician-patient communication [1,2]. As health care systems transition toward digital solutions, understanding patients’ ability to effectively engage with these tools becomes crucial, particularly in safety-net hospital settings, where digital health literacy barriers may impact care delivery and outcomes [3]. Digital health literacy, defined as “the ability to seek, find, understand, and appraise health information from electronic sources and apply the knowledge gained to addressing or solving a health problem” [4], plays a vital role in the successful implementation of digital health tools [2,5].

There is evidence suggesting that digital tools are effective in improving disease outcomes and health literacy [6-9]. Mobile health apps in the postoperative period have been correlated with earlier discharge, reduction of in-person follow-ups [10,11], promotion of rehabilitation [12], earlier detection of surgical complications [13], and improved communication between patients and health care professionals [14,15]. Beyond these clinical benefits, postoperative monitoring apps have shown potential to empower patients, providing autonomy over their own health and potentially improving satisfaction and recovery motivation [16-18].

However, it remains unclear what factors make these digital tools superior to human health navigators or resources available through simple internet searches [19], particularly in postoperative settings [20]. Despite the proliferation of digital health tools in postoperative care [21], limited research exists on digital health literacy and technology adoption readiness among safety-net hospital populations [22,23]. While studies demonstrate the efficacy of mobile apps in improving postoperative outcomes [24], these benefits presume a baseline level of digital health literacy that may not be universal across all patient populations, particularly in historically underserved communities [25].

In safety-net hospital settings, health literacy is shaped by factors such as educational level, language, and socioeconomic status, with lower health literacy levels linked to poorer health outcomes, reduced treatment adherence, and greater reliance on emergency care. Digital literacy, while related, is a distinct construct that poses additional barriers—particularly for older adults, non–English speakers, and individuals from lower socioeconomic backgrounds, who often face limited internet access and lack the digital skills necessary for navigating health care tools and systems. These separate constructs highlight a need for tailored tools to improve both forms of literacy [26].

The assumption that patients in safety-net hospitals face substantial barriers to digital health tool adoption has shaped both the development and implementation of digital interventions [23]. However, this assumption may not fully reflect current realities as smartphone ownership and digital technology use have become increasingly prevalent across socioeconomic groups [27,28]. As digital health tools continue to expand in postoperative care, understanding the actual digital health literacy levels and technology adoption readiness of safety-net hospital patients is essential for designing effective and equitable solutions [29].

In this study, we distinguished between 2 related but distinct constructs. *Digital health literacy* refers to patients’ perceived ability to seek, evaluate, and apply online health information, whereas *technology adoption readiness* reflects patients’ ability and confidence to engage with a specific digital health tool. Differentiating these constructs is critical as baseline literacy does not necessarily translate into successful interaction with or adoption of digital interventions.

In this paper, we present PocketDoc, a digital health application designed to support postoperative patient care, and use its evaluation to examine digital health literacy and technology adoption readiness in a safety-net hospital setting. We pursued 2 interrelated objectives. The primary aim was to assess digital health literacy levels among postoperative patients and health care providers. The secondary aim was to evaluate the usability of PocketDoc to determine whether a user-centered digital tool could be accessible to populations traditionally considered vulnerable to the digital divide.

These objectives are intentionally linked. Baseline digital health literacy provides essential context for interpreting usability findings, whereas usability outcomes inform whether patients are likely to adopt and meaningfully engage with digital health resources. By examining both constructs through a mixed methods approach—combining a modified eHealth Literacy Scale (eHEALS) assessment [30] with usability testing of a digital health prototype—we aimed to challenge prevailing assumptions about digital health literacy barriers and identify opportunities for more effective postoperative digital health tool implementation.

## Methods

### Study Design and Setting

This research was conducted at a large safety-net hospital in a Southern US state. We used a mixed methods approach combining quantitative assessments of digital health literacy with a qualitative usability testing of a digital health prototype.

### Study Population and Recruitment

Participants included 71 postoperative patients and 29 health care providers. Study inclusion criteria required participants to be adults (aged ≥18 years) who had had surgery within the previous year, were caretakers of someone who had had surgery in the previous year, or provided clinical care in the postsurgical period. Participants were not excluded based on health status, gender, race, or socioeconomic status, although patients with cognitive deficits or functional impairments preventing the use of digital devices were not recruited.

### Digital Health Literacy Assessment

Participants completed a modified version of the eHEALS, a validated instrument for measuring individuals’ combined knowledge, comfort, and perceived skills regarding finding, evaluating, and applying online health information [30]. The modified eHEALS included 7 of the original 8 items using its standard 5-point Likert scale (1=“not at all confident”; 5=“completely confident”). One question from the original eHEALS instrument, “I know how to use the health information I find on the internet to help me,” was inadvertently omitted during survey implementation. The modified 7-item scale demonstrated adequate internal consistency among the 71 patient participants (Cronbach α=0.77). We proceeded with analysis of the collected data while acknowledging this limitation in our interpretation of the results. This assessment captured perceived digital health literacy and did not evaluate task-based interaction with a specific technology.

### PocketDoc Development and Testing

**Figure 1.**
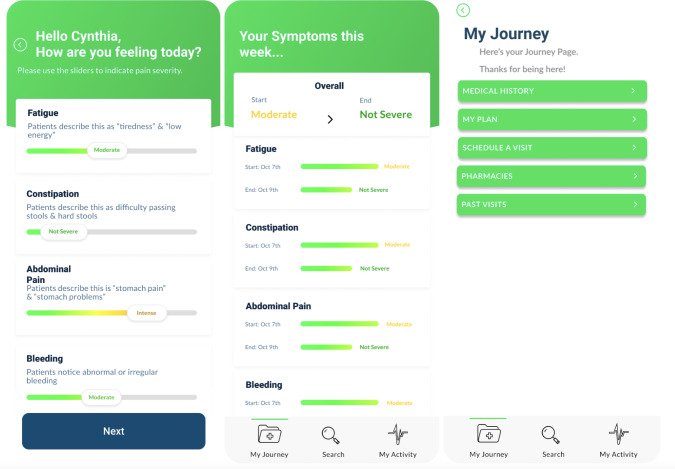
Select screenshots of the introduction page and “My Journey” tab of PocketDoc.

PocketDoc (Figure 1) is a comprehensive digital health tool designed to support patients during the postoperative period, particularly in historically underserved populations. Key functionality includes symptom tracking, medication management with reminders, secure messaging for communication with health care providers, and educational resources tailored to specific procedures. The platform also includes appointment scheduling, follow-up management, and real-time tools for assessing symptom burden, all aimed at enhancing recovery and improving patient outcomes. These features were designed to accommodate varying levels of digital literacy, with particular attention to intuitive navigation and clear information presentation. The interface incorporated evidence-based design principles for health care applications [31], including consistent layout patterns, clear call to action elements, and simplified medical terminology. The development process included multiple rounds of iterative design and input from design professionals, business consultants, and historically underserved patients through multiple rounds of feedback.

For the usability testing and posttask surveys, responses were collected using a 10-point Likert scale (1=“strongly disagree”; 10=“strongly agree”) to obtain granular insights into participants’ experiences with the digital health prototype. Usability testing and posttask surveys were used to assess technology adoption readiness and perceived usability rather than digital health literacy.

### Data Collection Procedures

Data collection occurred in 2 phases: the first phase included the demographic questionnaire, modified eHEALS assessment, and technology experience survey, and the second phase was the usability test, including standardized introductions to tasks, think-aloud protocols during task completion, and a posttask survey [32]. Sessions were conducted either in person or virtually via videoconference to accommodate participant availability, with each session lasting approximately 20 minutes.

### Data Analysis

Survey results were analyzed using the Kruskal-Wallis *H* test to examine differences in digital health literacy and tool adoption across geographic categories. Post hoc Dunn-Bonferroni tests were conducted to examine rural-suburban and rural-urban comparisons in terms of digital health literacy. The Spearman rank-order correlation was used for associations between patient-reported capabilities and use of health resources, as well as relationships between technology experience and tool adoption. Descriptive statistics were calculated for demographic and survey data. Exploratory linear regression analyses were conducted to examine whether technology experience (≥10 years vs <10 years) and overall digital health literacy were associated with patient confidence in their ability to use PocketDoc.

### Ethical Considerations

This research was approved by Emory University’s institutional review board in collaboration with a safety-net hospital in a southern US state (STUDY00004346). Written informed consent was obtained from all participants before taking part in the study. The informed consent process included a detailed explanation of study procedures, potential risks and benefits, the voluntary nature of participating, and the right to withdraw at any time. Participants were provided the opportunity to ask questions before providing consent. Participants received a US $20 gift card as compensation for their time. All data were deidentified before analysis.

## Results

### Participant Demographics

All 71 patients completed the modified eHEALS presurvey and subsequent assessments. Patient demographics are presented in Table 1. We found that there was diverse representation across age groups, with approximately half (n=33, 47%) between the ages of 18 and 34 years. Most participants came from urban (n=23, 32%) and suburban (n=43, 61%) environments, with limited rural representation (n=3, 4%). Most participants (n=41, 58%) had undergone surgery within the previous month, whereas 27% (n=19) had had surgery within the previous 6 months, and 14% (n=10) had had surgery within the previous year. Chronic conditions were reported by 58% (n=41) of the participants, indicating a population with ongoing health care needs. The patient sample was predominantly composed of Black individuals, reflecting the racial composition of the safety-net hospital population served.

**Table 1.** Demographic and clinical characteristics of postoperative patient participants (N=71).

Characteristic and category	Participants, n (%)
Survey completion	
eHEALS^a^ presurvey	71 (100)
PocketDoc postsurvey	71 (100)
Time when surgery was conducted	
Within the previous month	41 (57.7)
Within the previous 6 months	19 (26.8)
Within the previous year	10 (14.1)
Chronic conditions	
Yes	41 (57.7)
No	27 (38)
Number of chronic conditions (n=41)	
1	23 (56.1)
2	12 (29.3)
3	6 (14.6)
Age group (y)	
18-24	14 (19.7)
25-34	19 (26.8)
35-44	9 (12.7)
45-54	7 (9.9)
55-64	12 (16.9)
65-74	7 (9.9)
75-84	3 (4.2)
≥85	0 (0)

a eHEALS: eHealth Literacy Scale.

### Digital Health Literacy Levels

Analysis of the modified eHEALS survey (Table 2 and Multimedia Appendix 1) revealed that 69% (49/71) of patients reported high confidence (score of ≥3 on a 5-point scale) in finding health resources online (median 3, IQR 2-3). Furthermore, 61% (n=43) of participants expressed confidence in using the internet for health-related questions (median 3, IQR 2-3), and 63% (n=45) reported feeling capable of evaluating health resources (median 3, IQR 2-3). However, a notable drop occurred when examining confidence in health decision-making, with only 49% (n=35) feeling confident in using digital resources for making health decisions (median 2, IQR 2-3). Statistical analysis revealed that patients’ confidence in finding health resources was positively correlated with interface satisfaction (ρ=0.262, 95% CI 0.03‐0.47; *P*=.03). Similarly, patients’ confidence in using these resources showed a significant positive correlation with interface satisfaction (ρ=0.304, 95% CI 0.07‐0.52; *P*=.01).

**Table 2.** Item-level digital health literacy scores measured using the modified 7-item eHealth Literacy Scale (eHEALS).

eHEALS item	Score (1-5), median (IQR)
Evaluating resources	3 (2-3)
Knowing how to find resources	3 (2-3)
Using resources	3 (2-3)
Knowing about the existence of resources	3 (2-3.5)
Identifying the quality of resources	3 (2-3.5)
Knowing where to find resources	3 (2-4)
Making decisions with resources	2 (2-3)

### Technology Experience and Demographic Correlations

**Figure 2.**
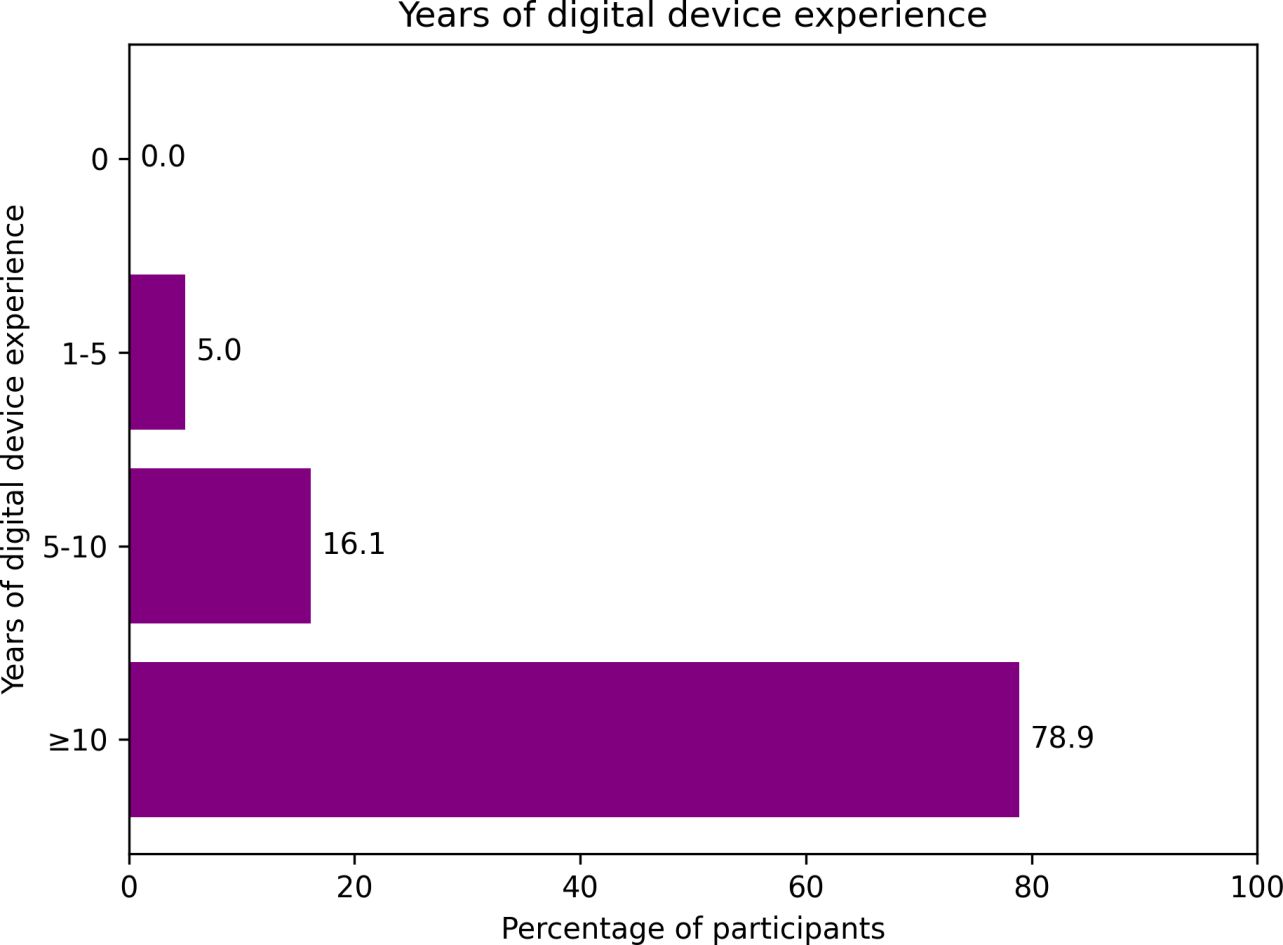
Years of digital device experience among postoperative patients (N=71).

Analysis of technology experience (Figure 2) revealed that 79% (56/71) of the participants reported 10 or more years of digital device experience, indicating substantial familiarity with technology within the study population. Statistical analysis demonstrated no significant correlation between digital literacy and age or educational level. However, geographic location emerged as a notable factor in digital literacy levels. The Kruskal-Wallis *H* test revealed significant differences in perceptions based on living environment (effect size=0.112; *P*=.05). Urban and suburban patients reported similarly high mean confidence scores (urban: mean 9/10, SD 1.33; suburban: mean 9/10, SD 1.19), whereas rural patients reported significantly lower scores (mean 7/10, SD 0.0). Post hoc Dunn-Bonferroni tests showed differences between rural-suburban (*P*=.081) and rural-urban comparisons (*P*=.080), which did not meet the significance threshold.

### Tool Adoption and Use Patterns

In the usability assessment of the digital health prototype (Tables3  4), participants demonstrated high levels of engagement and capability. Usability ratings across participants were high for ease of use (median 10, IQR 8-10), with task intuitiveness scoring highly at an average of 8.82 of 10 (median 10, IQR 8-10). Analysis revealed a significant correlation between prior technology exposure and perceived helpfulness of a digital health tool such as PocketDoc (ρ=0.230, 95% CI 0.01‐0.43; *P*=.04). Participants with ≥10 years of digital device experience showed particularly strong performance in information-finding tasks (median 9, IQR 7-10).

**Table 3.** Usability and technology adoption readiness of the PocketDoc prototype among postoperative patients (N=71).

Usability metric	Survey item	Score (1-10), median (IQR)
Ease of use	“I think PocketDoc was easy to use.”	10 (8-10)
Task scenario intuitiveness	“I think the Task Scenarios were intuitive to complete with PocketDoc.”	10 (8-10)
Interface satisfaction	“I like the interface of PocketDoc.”	9 (8-10)
Information findability	“I think my health information was presented to me in a way that was easy to find.”	9 (7-10)
Information organization	“I think my health information was presented to me in a way that was well organized.”	8 (7-10)
Compassionate wording	“I think my health information was presented to me in a way that was worded compassionately.”	10 (9-10)
Information summary	“I think my health information was presented to me in a way that was useful for summary.”	9 (7-10)
Health journey support	“I think PocketDoc would help me record my health journey after surgery.”	9 (7-10)
Patient confidence	“I am confident in my ability to use PocketDoc (as demonstrated).”	10 (9-10)

**Table 4.** Internal consistency of the modified 7-item eHealth Literacy Scale (eHEALS; Cronbach α=0.77).

Item	eHEALS item wording
1	“I know what health resources are available on the Internet.”
2	“I know where to find helpful health resources on the Internet.”
3	“I know how to find helpful health resources on the Internet.”
4	“I know how to use the Internet to answer my questions about health.”
6	“I have the skills I need to evaluate the health resources I find on the Internet.”
7	“I can tell high-quality health resources from low-quality health resources on the Internet.”
8	“I feel confident in using information from the Internet to make health decisions.”

Interestingly, participants’ ability to distinguish high-quality health resources showed a significant negative correlation with ease of finding information (ρ=−0.251, 95% CI −0.46 to −0.02; *P*=.04), whereas the correlation with information organization perception was marginally significant (ρ=−0.215, 95% CI −0.42 to 0.01; *P*=.07).

In an exploratory regression analysis examining predictors of confidence in ability to use PocketDoc, the overall model was statistically significant (*R*^2^=0.15; *P*=.004). Higher digital health literacy was independently associated with lower usability confidence (β=−0.31, 95% CI −0.50 to −0.12; *P*=.002), whereas technology experience (≥10 years vs <10 years) was not independently associated after accounting for literacy (β=0.36, 95% CI −0.19 to 0.91; *P*=.20).

## Discussion

### Principal Findings

Digital health literacy and technology adoption in safety-net hospital populations present a more nuanced picture than traditionally assumed. Prior research has suggested that patients in safety-net settings face barriers to digital health tool adoption due to limited access, lower digital literacy, and poor health literacy [26]. Our findings challenge the universality of these assumptions while highlighting specific areas in which targeted support may still be necessary for effective postoperative digital health implementation.

Importantly, digital health literacy and technology adoption readiness represent related but distinct constructs. The eHEALS captures patients’ perceived ability to seek, evaluate, and apply online health information broadly, whereas usability findings reflect patients’ interaction with a specific digital health tool within a defined clinical context. Distinguishing these constructs is critical for interpreting how baseline capabilities translate into real-world technology use.

In this study, relatively high levels of self-reported digital competency were observed, with 69% (49/71) of participants reporting high confidence in finding health resources online. This suggests that basic digital literacy barriers may be less universal among safety-net hospital patients than previously assumed [26]. These findings align with broader trends of increasing smartphone ownership and digital technology use across socioeconomic groups [27]. The high prevalence of substantial technology experience (n=56, 79% reporting ≥10 years of device use) further supports this shift.

Assessing digital health literacy alongside prototype usability provided important contextual insights. The relatively high baseline confidence in finding online health information helps frame the strong usability ratings observed for PocketDoc, including high scores for ease of use and task intuitiveness (median 10, IQR 8-10 in both cases). Together, these findings suggest that user-centered design approaches may facilitate accessibility across varying levels of digital health literacy.

A more granular pattern emerged when examining specific domains of digital health literacy. While many participants felt confident evaluating health resources (45/71, 63%) and using the internet for health-related questions (43/71, 61%), fewer reported confidence in using digital information for health decision-making (35/71, 49%). This pattern is consistent with prior work demonstrating that digital health literacy comprises multiple competencies, with decision-making often representing the greatest challenge [4,25,33,34].

The gap between general digital competency and confidence in health decision-making mirrors findings from recent studies highlighting the complexity of translating basic digital skills into applied health management [35,36]. This challenge may be particularly salient in postoperative care, where patients must interpret complex instructions and make time-sensitive decisions that carry immediate health implications [37].

Geographic differences in digital health literacy could not be robustly assessed due to the small rural sample size (n=3). Although rural participants demonstrated lower mean confidence scores than urban and suburban participants, these observations are exploratory and not suitable for inferential conclusions. Nonetheless, the observed pattern aligns with broader literature on rural-urban digital health disparities and underscores the need for future studies with adequately powered rural samples [35,38].

An additional insight emerged from the relationship between digital health literacy and usability perceptions. The negative association between critical evaluation skills and satisfaction with information organization (ρ=−0.251; *P*=.04) reflects a paradox observed in prior digital health research [39-41]. More digitally literate users may hold higher expectations for information presentation and usability rather than experiencing reduced capability.

This interpretation is further supported by exploratory regression analyses, which suggested that higher digital health literacy was associated with lower confidence in usability. Together, these findings highlight a key design challenge: digital health tools must accommodate users with varying levels of sophistication and expectations. Adaptive interfaces that provide both simplified overviews and optional advanced features may be particularly well suited for postoperative care contexts.

While this study also collected rich qualitative data through interviews and extended usability observations, this manuscript focuses on quantitative analyses and usability themes. A forthcoming manuscript will explore qualitative findings in greater depth to further contextualize patient experiences and perspectives. This approach was chosen to maintain focus on the study’s primary quantitative and usability outcomes.

### Limitations

Several limitations should be considered when interpreting these findings. Our use of a modified eHEALS, which inadvertently omitted question 5 (“I know how to use the health information I find on the internet to help me”), may have affected the comprehensiveness of our digital health literacy assessment. This modification constitutes use of a nonvalidated tool, and while the modified version demonstrated internal consistency (Cronbach α=0.77), this omission limits direct comparability with other eHEALS-based studies and potentially overestimates overall literacy levels.

Our sample included limited representation from rural areas (n=3), which precludes any meaningful conclusions about geographic disparities. Any observed differences between rural and both urban and suburban participants are reported as exploratory findings only and require replication in adequately powered samples. Relatedly, post hoc power for the geographic group comparison was low; thus, this study is underpowered to detect true rural–urban and suburban differences. This limitation is relevant given the differences we observed between both urban and suburban and rural participants.

This study’s reliance on self-reported measures of digital health literacy may not fully capture actual capabilities. While self-reported measures are widely used in digital health literacy research [42], they may be subject to social desirability bias and over- or underestimation of abilities. Our study may be subject to Berkson bias and self-selection bias as individuals with extremely low digital literacy or limited interest in digital tools may have been less likely to participate, and individuals with greater comfort with or interest in digital technologies may have been more likely to enroll.

Correlation analyses were exploratory and not adjusted for multiple testing, raising the possibility of type I error; therefore, the findings should be interpreted as hypothesis generating. Exploratory regression analyses were intentionally constrained to a small number of predictors due to sample size and ceiling effects in usability ratings and should not be interpreted as supporting causal inference.

### Conclusions

This study advances our understanding of the evolving landscape of digital readiness among postoperative patients in safety-net settings. While participants reported substantial experience with digital devices, the data highlight a persistent challenge: translating general digital familiarity into confidence in managing personal health decisions. This gap suggests that, even when access and basic skills are present, patients may still struggle to apply digital tools meaningfully in complex clinical contexts such as postoperative care. Moreover, preliminary geographic trends point to location-based factors rather than traditional demographic variables as potential influences on confidence and engagement with digital health tools. These insights underscore the importance of designing tools that go beyond increasing access or general literacy, instead emphasizing support for decision-making and personalization based on patients’ lived environments. Addressing these nuanced barriers may be key to optimizing digital health strategies and ensuring equitable postoperative support across diverse care settings.

## Supplementary material

10.2196/75496 Multimedia Appendix 1 Patient-reported digital health literacy scores by domain.

## Abbreviations

eHEALS: eHealth Literacy Scale

## References

[1] Zhang J, Ge Y, Yang M, Ivers R, Webster R, Tian M (2021). The role of digital health for post-surgery care of older patients with hip fracture: a scoping review. JMIR Preprints.

[2] He M, Chen M, Ji Y, Lu G (2024). Effectiveness of smartphone app-based interventions after surgery on quality of recovery among cancer patients: a systematic review and meta-analysis. Ann Med.

[3] Kemp E, Trigg J, Beatty L (2021). Health literacy, digital health literacy and the implementation of digital health technologies in cancer care: the need for a strategic approach. Health Promot J Austr.

[4] Conard S (2019). Best practices in digital health literacy. Int J Cardiol.

[5] Campanozzi LL, Gibelli F, Bailo P, Nittari G, Sirignano A, Ricci G (2023). The role of digital literacy in achieving health equity in the third millennium society: a literature review. Front Public Health.

[6] Guhl E, Althouse AD, Pusateri AM (2020). The atrial fibrillation health literacy information technology trial: pilot trial of a mobile health app for atrial fibrillation. JMIR Cardio.

[7] Hendawi R, Alian S, Li J (2022). A smart mobile app to simplify medical documents and improve health literacy: system design and feasibility validation. JMIR Form Res.

[8] McKay FH, Cheng C, Wright A, Shill J, Stephens H, Uccellini M (2018). Evaluating mobile phone applications for health behaviour change: a systematic review. J Telemed Telecare.

[9] Wiljer D, Shi J, Lo B (2020). Effects of a mobile and web app (thought spot) on mental health help-seeking among college and university students: randomized controlled trial. J Med Internet Res.

[10] Armstrong KA, Coyte PC, Brown M, Beber B, Semple JL (2017). Effect of home monitoring via mobile app on the number of in-person visits following ambulatory surgery: a randomized clinical trial. JAMA Surg.

[11] Lee L, Eustache J, Baldini G (2022). Enhanced recovery 2.0 - same day discharge with mobile app follow-up after minimally invasive colorectal surgery. Ann Surg.

[12] Belarmino A, Walsh R, Alshak M, Patel N, Wu R, Hu JC (2019). Feasibility of a mobile health application to monitor recovery and patient-reported outcomes after robot-assisted radical prostatectomy. Eur Urol Oncol.

[13] Hee Hwang J, Mun GH (2012). An evolution of communication in postoperative free flap monitoring: using a smartphone and mobile messenger application. Plast Reconstr Surg.

[14] Lu K, Marino NE, Russell D (2018). Use of short message service and smartphone applications in the management of surgical patients: a systematic review. Telemed J E Health.

[15] Abelson JS, Kaufman E, Symer M, Peters A, Charlson M, Yeo H (2017). Barriers and benefits to using mobile health technology after operation: a qualitative study. Surgery.

[16] Semple JL, Sharpe S, Murnaghan ML, Theodoropoulos J, Metcalfe KA (2015). Using a mobile app for monitoring post-operative quality of recovery of patients at home: a feasibility study. JMIR Mhealth Uhealth.

[17] Gunter R, Fernandes-Taylor S, Mahnke A (2016). Evaluating patient usability of an image-based mobile health platform for postoperative wound monitoring. JMIR Mhealth Uhealth.

[18] Temple-Oberle C, Yakaback S, Webb C, Assadzadeh GE, Nelson G (2023). Effect of smartphone app postoperative home monitoring after oncologic surgery on quality of recovery: a randomized clinical trial. JAMA Surg.

[19] Bardus M, van Beurden SB, Smith JR, Abraham C (2016). A review and content analysis of engagement, functionality, aesthetics, information quality, and change techniques in the most popular commercial apps for weight management. Int J Behav Nutr Phys Act.

[20] Spreadbury JH, Young A, Kipps CM (2022). A comprehensive literature search of digital health technology use in neurological conditions: review of digital tools to promote self-management and support. J Med Internet Res.

[21] Robinson A, Oksuz U, Slight R, Slight S, Husband A (2020). Digital and mobile technologies to promote physical health behavior change and provide psychological support for patients undergoing elective surgery: meta-ethnography and systematic review. JMIR Mhealth Uhealth.

[22] Yao R, Zhang W, Evans R, Cao G, Rui T, Shen L (2022). Inequities in health care services caused by the adoption of digital health technologies: scoping review. J Med Internet Res.

[23] Tieu L, Sarkar U, Schillinger D (2015). Barriers and facilitators to online portal use among patients and caregivers in a safety net health care system: a qualitative study. J Med Internet Res.

[24] Dawes AJ, Lin AY, Varghese C, Russell MM, Lin AY (2021). Mobile health technology for remote home monitoring after surgery: a meta-analysis. Br J Surg.

[25] Smith B, Magnani JW (2019). New technologies, new disparities: the intersection of electronic health and digital health literacy. Int J Cardiol.

[26] Burke J, Higgins MG, Vemuru SR (2024). Self-reported health literacy, digital literacy, and barriers to accessing care at a safety net breast surgical oncology clinic. medRxiv.

[27] Tsetsi E, Rains SA (2017). Smartphone internet access and use: extending the digital divide and usage gap. Mob Media Commun.

[28] Marler W (2018). Mobile phones and inequality: findings, trends, and future directions. New Media Soc.

[29] Lee WL, Lim ZJ, Tang LY, Yahya NA, Varathan KD, Ludin SM (2021). Patients’ technology readiness and eHealth literacy: implications for adoption and deployment of eHealth in the COVID-19 era and beyond. Comput Inform Nurs.

[30] Norman CD, Skinner HA (2006). eHEALS: the eHealth Literacy Scale. J Med Internet Res.

[31] Shneiderman B, Plaisant C, Cohen M, Jacobs S (2010). Designing the User Interface: Strategies for Effective Human-Computer Interaction.

[32] Nielsen J (1993). Usability Engineering.

[33] van der Heide I, Poureslami I, Mitic W, Shum J, Rootman I, FitzGerald JM (2018). Health literacy in chronic disease management: a matter of interaction. J Clin Epidemiol.

[34] Holmes-Rovner M, Kroll J, Schmitt N (1996). Patient satisfaction with health care decisions: the satisfaction with decision scale. Med Decis Making.

[35] Arias López MD, Ong BA, Borrat Frigola X (2023). Digital literacy as a new determinant of health: a scoping review. PLOS Digit Health.

[36] Mainz A, Nitsche J, Weirauch V, Meister S (2024). Measuring the digital competence of health professionals: scoping review. JMIR Med Educ.

[37] Esper SA, Holder-Murray J, Meister KA (2024). A novel digital health platform with health coaches to optimize surgical patients: feasibility study at a large academic health system. JMIR Perioper Med.

[38] Shiferaw KB, Tilahun BC, Endehabtu BF (2020). Healthcare providers’ digital competency: a cross-sectional survey in a low-income country setting. BMC Health Serv Res.

[39] Liu P, Yeh LL, Wang JY, Lee ST (2020). Relationship between levels of digital health literacy based on the Taiwan digital health literacy assessment and accurate assessment of online health information: cross-sectional questionnaire study. J Med Internet Res.

[40] Kinney AP, Sankaranarayanan B (2021). Effects of patient portal use on patient satisfaction: survey and partial least squares analysis. J Med Internet Res.

[41] Zhao BY, Chen MR, Lin R, Yan YJ, Li H (2024). Influence of information anxiety on core competency of registered nurses: mediating effect of digital health literacy. BMC Nurs.

[42] van der Vaart R, Drossaert C (2017). Development of the digital health literacy instrument: measuring a broad spectrum of health 1.0 and health 2.0 skills. J Med Internet Res.

