# Supplementary figures and images for: Digital Health Literacy and Tool Adoption in Postoperative Care in a Safety-Net Hospital Population: Mixed Methods Study
Source: JMIR Hum Factors. 2026 Feb 23;13:e75496. doi: 10.2196/75496 (PMC12928685; doi:10.2196/75496)

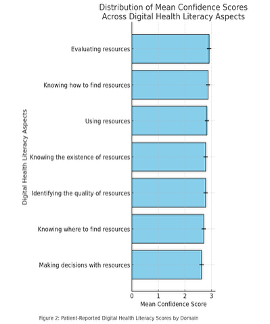

Supplement: Multimedia Appendix 1 [file humanfactors-v13-e75496-s001.png]
